# E-cigarette Use Among Middle and High School Students — United States, 2020

**DOI:** 10.15585/mmwr.mm6937e1

**Published:** 2020-09-18

**Authors:** Teresa W. Wang, Linda J. Neff, Eunice Park-Lee, Chunfeng Ren, Karen A. Cullen, Brian A. King

**Affiliations:** ^1^Office on Smoking and Health, National Center for Chronic Disease Prevention and Health Promotion, CDC; ^2^Center for Tobacco Products, Food and Drug Administration, Silver Spring, Maryland.

The use of any tobacco product by youths is unsafe, including electronic cigarettes (e-cigarettes) *(1)*. Most e-cigarettes contain nicotine, which is highly addictive, can harm the developing adolescent brain, and can increase risk for future addiction to other drugs (*1*). E-cigarette use has increased considerably among U.S. youths since 2011 (*1*,*2*). Multiple factors have contributed to this increase, including youth-appealing flavors and product innovations (*1*–*3*). Amid the widespread use of e-cigarettes and popularity of certain products among youths, on February 6, 2020, the Food and Drug Administration (FDA) implemented a policy prioritizing enforcement against the manufacture, distribution, and sale of certain unauthorized flavored prefilled pod or cartridge-based e-cigarettes (excluding tobacco or menthol).*

CDC and FDA analyzed nationally representative data from the 2020 National Youth Tobacco Survey (NYTS),^†^ a cross-sectional, school-based, self-administered survey of U.S. middle school (grades 6–8) and high school (grades 9–12) students conducted during January 16–March 16, 2020.^§^ The NYTS study protocol was approved by the CDC institutional review board. Current (past 30-day) e-cigarette use was assessed, overall and by device^¶^ and flavor** type. Weighted prevalence estimates and population totals^††^ were calculated. Analyses were conducted using SAS-callable SUDAAN (version 11.0.3; RTI International).

In 2020, 19.6% of high school students (3.02 million) and 4.7% of middle school students (550,000) reported current e-cigarette use. Among current e-cigarette users, 38.9% of high school students and 20.0% of middle school students reported using e-cigarettes on 20 or more of the past 30 days; 22.5% of high school users and 9.4% of middle school users reported daily use. Among all current e-cigarette users, 82.9% used flavored e-cigarettes, including 84.7% of high school users (2.53 million) and 73.9% of middle school users (400,000).

Among high school current e-cigarette users, the most commonly used device type was prefilled pods or cartridges (48.5%; 1.45 million), followed by disposables (26.5%; 790,000), and tanks (14.8%; 440,000). Among middle school current e-cigarette users, the most commonly used device type was prefilled pods or cartridges (41.3%; 220,000), followed by tanks (21.5%; 110,000), and disposables (15.2%; 80,000).

Among high school students who currently used any type of flavored e-cigarettes, the most commonly used flavor types were fruit (73.1%; 1.83 million); mint (55.8%; 1.39 million); menthol (37.0%; 920,000); and candy, desserts, or other sweets (36.4%; 910,000). Among middle school students who currently used any type of flavored e-cigarettes, the most commonly used flavor types were fruit (75.6%; 290,000); candy, desserts, or other sweets (47.2%; 180,000); mint (46.5%; 180,000); and menthol (23.5%; 90,000).

Among current users of flavored prefilled pods or cartridges, the most commonly used flavor types were fruit (66.0%; 920,000); mint (57.5%; 800,000); menthol (44.5%; 620,000); and candy, desserts, or other sweets (35.6%; 490,000) (Figure). Among current users of flavored disposable e-cigarettes, the most commonly used flavor types were fruit (82.7%; 650,000), mint (51.9%; 410,000); candy, desserts, or other sweets (41.7%; 330,000); and menthol (23.3%; 180,000).

**FIGURE:**
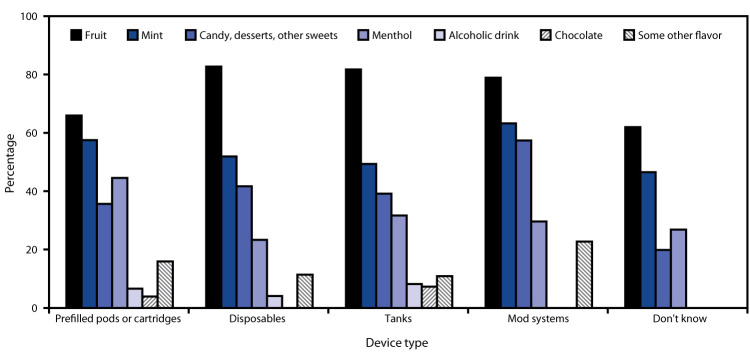
Percentage of flavor types used by current (past 30-day) flavored e-cigarette users among U.S. middle and high school students,* by device type^†,^^§^ — National Youth Tobacco Survey, United States, 2020 * Flavor type use among current (past 30-day) users of flavored e-cigarettes was determined by answers to the question “What flavors were the e-cigarettes that you have used in the past 30 days? (Select one or more).” Response options were “menthol,” “mint,” “clove or spice,” “fruit,” “chocolate,” “alcoholic drinks (such as wine, cognac, margarita, or other cocktails),” “candy, desserts, or other sweets,” and “some other flavor not listed here” (write-in responses were not assessed). Data for “clove or spice” are not shown because of statistically unreliable estimates due to unweighted denominator <50 or relative standard error >30% across all device types. ^†^ Device type use among current e-cigarette users was determined by answers to the question “Which of the following best describes the type of e-cigarette you have used in the past 30 days? If you have used more than one type, please think about the one you use most often.” Response options were “a disposable e-cigarette,” “an e-cigarette that uses pre-filled pods or cartridges (e.g., JUUL),” “an e-cigarette with a tank that you refill with liquids,” “a mod system (an e-cigarette that can be customized by the user with their own combination of batteries or other parts),” and “I don’t know the type.” ^§^ The following data were statistically unreliable and not shown due to unweighted denominator <50 or relative standard error >30%: use of chocolate flavor types among current flavored e-cigarette users of disposable e-cigarettes, mod systems, or those who reported “I don’t know the type” for device type; alcoholic drink flavor types among current flavored e-cigarette users of mod systems or those who reported “I don’t know the type” for device type; and “some other flavor” among current flavored e-cigarette users who reported “I don’t know the type” for device type.

In 2020, approximately one in five high school students and one in 20 middle school students currently used e-cigarettes. By comparison, in 2019, 27.5% of high school students (4.11 million) and 10.5% of middle school students (1.24 million) reported current e-cigarette use (*2*). Although these data reflect a decline in current e-cigarette use since 2019, 3.6 million U.S. youths still currently used e-cigarettes in 2020, and among current users, more than eight in 10 reported using flavored e-cigarettes. 

Consistent with 2019, prefilled pods or cartridges were the most commonly used device type in 2020; however, during 2019–2020, disposable e-cigarette use increased approximately 1,000% (from 2.4% to 26.5%) among high school current e-cigarette users and approximately 400% (from 3.0% to 15.2%) among middle school current e-cigarette users. Although use of fruit flavored e-cigarettes was common among users in 2020, findings also suggest prominent menthol e-cigarette use, including among nearly one half of flavored prefilled pod or cartridge users and one quarter of flavored disposable product users.

Comprehensive implementation of evidence-based strategies at the national, state, and local levels, in coordination with FDA regulation, can prevent and reduce youth tobacco product use (*1*,*4*,*5*). Strategies to address factors driving youth e-cigarette use are particularly critical. In addition to FDA’s enforcement policy that prohibits the sale of prefilled pod or cartridge-based e-cigarettes in any flavor other than tobacco or menthol, several states and communities have restricted all flavored e-cigarette sales, including menthol.^§§^

## Footnotes

*
https://www.fda.gov/news-events/press-announcements/fda-finalizes-enforcement-policy-unauthorized-flavored-cartridge-based-e-cigarettes-appeal-children .

§ The data collection timeline was truncated because of widespread school closures during the coronavirus disease 2019 pandemic.

¶ Device type use among current e-cigarette users was determined by answers to the question “Which of the following best describes the type of e-cigarette you have used in the past 30 days? If you have used more than one type, please think about the one you use most often.” Response options were “a disposable e-cigarette,” “an e-cigarette that uses pre-filled pods or cartridges (e.g., JUUL),” “an e-cigarette with a tank that you refill with liquids,” “a mod system (an e-cigarette that can be customized by the user with their own combination of batteries or other parts),” and “I don’t know the type.”

** Flavored e-cigarette use among current e-cigarette users was determined by answers to the question “Were any of the e-cigarettes that you used in the past 30 days flavored to taste like menthol, mint, clove or spice, alcohol (wine, cognac), candy, fruit, chocolate, or any other flavor?” Response options were “yes,” “no,” and “don’t know.” Flavor type use among current e-cigarette users who reported flavored e-cigarette use was determined by answers to the question “What flavors were the e-cigarettes that you have used in the past 30 days? (Select one or more).” Response options were: “menthol,” “mint,” “clove or spice,” “fruit,” “chocolate,” “alcoholic drinks (such as wine, cognac, margarita, or other cocktails),” “candy, desserts, or other sweets,” and “some other flavor not listed here” (write-in responses were not assessed).

†† Weighted population estimates are rounded down to the nearest 10,000 students.

§§ https://www.tobaccofreekids.org/assets/factsheets/0398.pdf.
